# Gender-specific association between AST/ALT ratio and 365-day mortality in patients with severe acute pancreatitis: A retrospective cohort study based on the MIMIC-IV database

**DOI:** 10.1097/MD.0000000000048324

**Published:** 2026-05-01

**Authors:** Zhimin Dong, Dasheng Dang

**Affiliations:** a Department of Pharmacy, The First Affiliated Hospital of Xi’an Jiaotong University, Xi’an, China; b Department of Pharmacy, Northern Theater General Hospital of The People’s Liberation Army, Shenyang, China.

**Keywords:** acute pancreatitis, aspartate aminotransferase to alanine aminotransferase ratio, intensive care unit, MIMIC-IV, mortality

## Abstract

Patients with acute pancreatitis (AP) in the intensive care unit experience high complication and mortality rates, making early prognosis prediction crucial. While the aspartate aminotransferase to alanine aminotransferase (AST/ALT) ratio is correlates with prognosis in various diseases, its association with mortality in severe AP remains uncertain. AP patients were identified using the Medical Information Mart for Intensive Care-IV database. Cox proportional hazard regression models examined the link between the AST/ALT ratio and 365-day mortality. Kaplan–Meier survival curve analysis was conducted to assess patient survival across AST/ALT ratio quartiles. restricted cubic splines analysis determined the relationship curve between 365-day mortality and the AST/ALT ratio level. We also conducted subgroup analyses to assess the robustness of the findings. A total of 817 patients were included in this retrospective cohort study. After adjusting for all covariates, the Cox regression model showed that the AST/ALT ratio was positively associated with 365-day mortality in patients with AP (hazard ratio: 1.33, 95% confidence interval: 1.16–1.52). When the AST/ALT ratio was divided into 4 groups based on quartiles, participants in the *Q*4 group had a significantly higher mortality compared with *Q*1 (hazard ratio:2.11, 95% confidence interval: 1.30–3.40). The Kaplan–Meier curves showed that patients in the *Q*1 group had the lowest mortality (log-rank *P* < .001). The restricted cubic splines curve indicated a linear relationship between the AST/ALT ratio and mortality in AP patients (*P* for nonlinear > .05). Subgroup analyses further demonstrated that this association was more pronounced in men. Finally, the AST/ALT ratio improved the predictive performance of sequential organ failure assessment score for mortality in patients with AP. In critically ill AP patients, a higher AST/ALT ratio on admission was associated with greater 365-day mortality, especially in males. It is suggested that the AST/ALT ratio can be used as a simple and effective prognostic marker for patients with severe AP.

## 1. Introduction

Acute pancreatitis (AP) is an inflammatory disorder of the pancreas characterized by a clinical spectrum ranging from mild to severe presentations, typically manifesting as sudden-onset epigastric pain.^[1]^ Most patients demonstrate indolent disease progression and respond well to intravenous fluid resuscitation and supportive measures, as pancreatic autodigestion is generally self-limiting. However, 10% to 20% of cases progress to moderate or severe AP, characterized by pancreatic/peripancreatic necrosis and potential multiorgan dysfunction syndrome (MODS), with associated mortality rates reaching 20% to 30%.^[2]^ Rising global AP incidence correlates with ~10% of patients requiring intensive care unit (ICU) admission due to life-threatening complications.^[3]^ Prognostic determinants include clinician assessment, computed tomography findings, elevated body mass index, genetic predisposition, and multifactorial clinical scoring systems.^[4]^ Pancreatitis severity is evaluated using methods such as the Ranson criteria, which assesses 11 clinical and laboratory parameters over 48 hours; the bedside index for severity in acute pancreatitis, which evaluates 5 parameters within 24 hours; and the acute physiology and chronic health evaluation II, which includes age, chronic health status, and 12 acute physiological markers. Additionally, the Balthazar classification and the sequential organ failure assessment (SOFA) are utilized, all of which necessitate many clinical indicators and time.^[5-7]^ Notably, emerging biomarkers such as C-reactive protein, procalcitonin, and interleukin-6 have shown enhanced predictive value for disease severity.^[8]^ Given the rapid clinical deterioration, substantial mortality burden, and resource-intensive monitoring requirements in critical care settings, developing simplified rapid-assessment biomarkers for AP severity remains an urgent unmet clinical need.^[9,10]^

Aspartate aminotransferase (AST) is a mitochondrial and cytosolic enzyme ubiquitously expressed across tissues, functioning as a sensitive marker of oxidative stress-induced mitochondrial injury. Conversely, alanine aminotransferase (ALT) demonstrates predominant hepatic cytosolic localization, serving as a hepatocyte-specific indicator of liver parenchymal damage.^[11,12]^ The AST/ALT ratio not only reflects hepatic fibrotic progression and functional impairment but also correlates with systemic pathological processes, including oxidative stress and inflammatory cascades.^[13,14]^ The AST/ALT ratio has emerged as a clinically significant biomarker with prognostic value across diverse clinical settings. It has been associated with adverse outcomes in cardiovascular diseases (including diabetes, insulin resistance, obesity, and hypertension), multi-organ failure, and chronic kidney disease.^[15-18]^ Additionally, accumulating evidence links the AST/ALT ratio to poor prognosis in conditions such as cancer, sepsis, cirrhosis, and COVID-19.^[19-22]^ Previous studies have demonstrated that both ALT and AST levels are positively associated with the severity of pancreatitis. Notably, a threefold increase in ALT within 48 hours of AP onset has been identified as a potential indicator of biliary etiology, which accounts for 30% to 70% of pancreatitis cases.^[23-25]^ However, the relationship between the AST/ALT ratio and AP remains incompletely understood and warrants further investigation. Furthermore, previous studies have demonstrated that ALT and AST levels exhibit gender differences.^[26]^ This leads us to hypothesize that the prognostic value of the AST/ALT ratio in AP may vary by gender.

The present study investigated the association between the AST/ALT ratio and 365-day mortality in critically ill patients with AP. We hypothesized that an elevated AST/ALT ratio at admission may be independently associated with increased 365-day mortality in this patient population. Elucidating this relationship could enhance risk stratification models and facilitate the development of more personalized therapeutic approaches, ultimately improving clinical outcomes for critically ill patients with AP.

## 2. Materials and methods

### 2.1. Data source

This research utilized data solely from the publicly accessible Medical Information Mart for Intensive Care-IV (MIMIC-IV) database. The database received approval from the Institutional Review Boards of both the Massachusetts Institute of Technology and Beth Israel Deaconess Medical Centre. The Ethical Committee of the Beth Israel Deaconess Medical Centre waived the need for informed consent as all data were de-identified to ensure patient privacy. The dataset encompasses clinical information from thousands of individuals, covering the period from 2008 to 2019.

### 2.2. Study population

**Figure 1.**
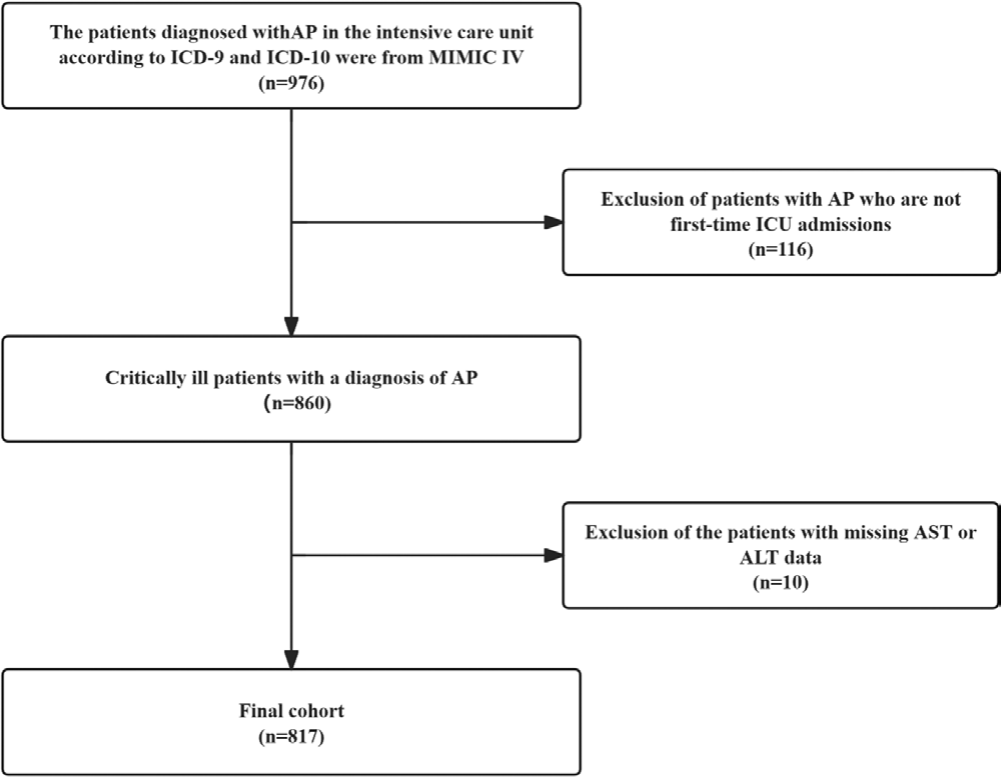
Flowchart of subject screening. ALT = alanine aminotransferase, AP = acute pancreatitis, AST = aspartate aminotransferase, ICU = intensive care unit, MIMIC IV = , Medical Information Mart for Intensive Care-IV.

We defaulted AP patients admitted to the ICU as severe cases. This pragmatic definition is based on the clinical reality that ICU admission for AP typically indicates significant disease severity requiring critical care support, consistent with the Revised Atlanta Classification’s emphasis on organ failure as the key determinant of severity.^[27]^ This study examined adult patients (≥18 years) diagnosed with AP, who had a recorded AST/ALT ratio within 24 hours of ICU admission during hospitalization. Definitions of patients with AP are based on ICD-9 or ICD-10. We defaulted AP patients admitted to the ICU as severe cases. The study began by screening 976 patients with AP, excluding patients not admitted to the ICU for the first time (n = 116) or missing predefined AST/ALT data (n = 43), resulting in a final cohort of 817 patients. Figure 1 outlines the research’s inclusion and exclusion process.

### 2.3. Data extraction

Data was carefully extracted from the MIMIC-IV database using PostgreSQL version 13.0 on February 16, 2025. Based on previous studies and clinical experience, the following patient characteristics were collected^[28-31]^: demographic details: age, and gender. Vital signs: heart rate, systolic blood pressure, diastolic blood pressure, mean blood pressure, respiratory rate, temperature, and saturation of peripheral oxygen. Comorbidities: acute kidney injury (AKI), hypertension, diabetes, myocardial infarction, malignant tumors, stroke, atrial fibrillation, obesity, respiratory failure, sepsis, gallstones, and liver disease (including hepatitis, cirrhosis and alcoholic liver disease). The initial laboratory values recorded within 24 hours of ICU admission: white blood cell, red blood cell (RBC), hemoglobin, red cell distribution width (RDW), platelets, albumin, anion gap, calcium, chloride, potassium, sodium, glucose, prothrombin time (PT), partial thromboplastin time (PTT), aspartate aminotransferase (ALT), alanine aminotransferase (AST), creatinine, and urea nitrogen. Severity scoring system: SOFA score, acute physiology score III (APS III), simplified acute physiology score II (SAPS II), and Oxford Acute Severity of Illness Score (OASIS). Drug use: beta-blockers, metformin, octreotide, statin, and vasopressin. Treatment interventions: continuous renal replacement therapy, and ventilation. Length of ICU stay and hospitalization, along with ICU, in-hospital, and 365-day mortality.

We removed variables with more than 20% missing values; for variables with <20% missing values, we interpolated using the Random Forest method.

### 2.4. Definition outcomes

The primary endpoint was to assess the all-cause mortality 365 days post-admission. Mortality data were obtained from ICU admission records and in-hospital and out-of-hospital date-of-death records.

### 2.5. Statistical analysis

Differences between deceased and surviving patients were first analyzed, and then AST/ALT ratios were divided into quartiles (*Q*1: 0.22–0.90; *Q*2: 0.90–1.33; *Q*3: 1.33–1.98; *Q*4: 1.98–9.20) based on historical studies.^[32,33]^ Categorical variables were expressed as percentages, and the χ^2^ test or Fisher’s exact probability method was used to analyze them. Continuous variables were tested for differences between groups using a rank-sum test and are expressed as the median and interquartile range. Kaplan–Meier curves were used to analyze 365-day survival probabilities for patients with AP across different AST/ALT levels. Covariates that entered the multivariate Cox regression model were selected by screening for variables that differed between prognostic outcomes, covariates with covariate diagnostic variance inflation factor <5, and known risk factors for AP. We developed 3 models: model 1 (unadjusted), model 2 (adjusted for age, gender, and weight), and model 3 (adjusted for all other variables). A restricted cubic spline (RCS) was employed to examine the potential nonlinear association between the AST/ALT ratio and 365-day mortality. Furthermore, the relationship between the AST/ALT ratio and critically ill AP patients was analyzed across various population subgroups. Finally, we used the receiver operating characteristic curve as well as the area under the curve to compare whether AST/ALT ratio increases the predictive power of SOFA for the risk of death in patients with AP.

Statistical analyses were conducted using R software (v4.3.1) with a significant level set at *P* < .05. Our statistical analysis used the R packages “survival,” “rms,” “survminer” for survival modeling, in addition to the “car,” “tableone” and “tidyverse” packages.

## 3. Results

### 3.1. Baseline characteristics

The study included 817 AP patients, with a median age of 59 years, of whom 59.00% were males. Table 1 presents a comparison of baseline characteristics between survivors and non-survivors. Compared to survivors, non-survivors were older. They also showed more derangements in laboratory values (e.g., higher RDW, potassium, anion gap, PT, PTT, AST/ALT ratio, urea nitrogen, creatinine, and lower RBC count, hemoglobin, albumin) and more abnormal vital signs (lower blood pressures and body temperature). Severity scores (SOFA, APS III, SAPS II, OASIS) were higher in non-survivors, consistent with their greater incidence of acute complications like AKI, malignant tumors, atrial fibrillation, respiratory failure, and sepsis. Non-survivors also required continuous renal replacement therapy, vasopressors, and octreotide more frequently.

**Table 1:** Baseline characteristics of the AP population according to 365-day mortality.

	Total (n = 817)	Survival (n = 644)	Non-survival (n = 173)	*P*
Age, yr	59.00 (47.00, 73.00)	57.00 (45.00, 70.00)	70.00 (56.00, 81.00)	<.001
Gender, n (%)				.291
Female	335 (41.00)	258 (40.06)	77 (44.51)	
Male	482 (59.00)	386 (59.94)	96 (55.49)	
Weight, kg	83.10 (70.70, 99.00)	83.32 (71.18, 101.03)	81.20 (69.00, 94.20)	.082
WBC, K/µL	12.50 (8.50, 17.90)	12.15 (8.38, 17.42)	13.60 (9.00, 19.00)	.071
RBC, m/µL	3.67 (3.15, 4.20)	3.73 (3.20, 4.21)	3.50 (2.96, 4.08)	.004
Platelet, K/µL	185.00 (129.00, 258.00)	188.00 (133.75, 256.50)	171.00 (114.00, 260.00)	.071
Hemoglobin, g/dL	11.20 (9.70, 12.80)	11.30 (9.80, 12.90)	10.60 (9.20, 12.30)	<.001
RDW, %	14.50 (13.60, 15.80)	14.40 (13.50, 15.40)	15.50 (14.20, 17.10)	<.001
Albumin, g/dL	3.00 (2.60, 3.30)	3.02 (2.70, 3.40)	2.80 (2.30, 3.20)	<.001
Sodium, mmol/L	138.00 (135.00, 142.00)	138.00 (135.00, 141.00)	138.00 (135.00, 142.00)	.318
Potassium, mmol/L	4.00 (3.60, 4.50)	4.00 (3.60, 4.40)	4.10 (3.70, 4.80)	.004
Calcium, mg/dL	8.00 (7.40, 8.50)	8.00 (7.40, 8.50)	8.00 (7.30, 8.50)	.528
Chloride, mmol/L	104.00 (100.00, 109.00)	104.00 (100.00, 109.00)	105.00 (100.00, 110.00)	.309
Glucose, mg/dL	125.00 (102.00, 168.00)	124.00 (102.00, 169.00)	128.00 (101.00, 167.00)	.669
Anion gap, mmol/L	15.00 (13.00, 18.00)	15.00 (13.00, 18.00)	17.00 (14.00, 21.00)	<.001
PT, s	14.40 (12.90, 16.70)	14.10 (12.90, 16.00)	15.70 (13.70, 18.90)	<.001
PTT, s	30.90 (27.30, 35.90)	30.40 (27.20, 34.62)	32.80 (28.60, 42.00)	<.001
AST/ALT ratio	1.33 (0.90, 1.98)	1.28 (0.86, 1.88)	1.54 (1.02, 2.62)	<.001
Urea nitrogen, mg/dL	19.00 (12.00, 33.00)	17.00 (11.00, 27.00)	32.00 (19.00, 50.00)	<.001
Creatinine, mg/dL	1.00 (0.70, 1.70)	0.90 (0.70, 1.50)	1.50 (0.90, 2.40)	<.001
SOFA score	5.00 (2.00, 8.00)	4.00 (2.00, 7.00)	7.00 (4.00, 11.00)	<.001
APS III score	46.00 (33.00, 64.00)	42.50 (31.00, 57.00)	65.00 (45.00, 85.00)	<.001
SAPS II score	34.00 (24.00, 45.00)	31.00 (22.00, 42.00)	46.00 (36.00, 59.00)	<.001
OASIS score	32.00 (26.00, 39.00)	31.00 (25.00, 37.00)	38.00 (30.00, 45.00)	<.001
Heart rate, beats/min	97.00 (82.00, 114.00)	98.00 (82.00, 115.00)	95.00 (83.00, 112.00)	.473
SBP, mm Hg	126.00 (109.00, 144.00)	129.00 (112.00, 145.00)	116.00 (102.00, 142.00)	<.001
DBP, mm Hg	72.00 (59.00, 85.00)	74.00 (62.00, 87.00)	65.00 (53.00, 79.00)	<.001
MBP, mm Hg	85.00 (72.00, 99.00)	86.00 (74.00, 100.00)	80.00 (66.00, 91.00)	<.001
Respiratory rate, breath/minute	20.00 (16.00, 25.00)	20.00 (16.00, 24.25)	20.00 (17.00, 25.00)	.454
SpO_2_, %	96.00 (94.00, 99.00)	96.00 (94.00, 99.00)	97.00 (94.00, 99.00)	.126
Temperature, °C	36.89 (36.56, 37.28)	36.94 (36.61, 37.39)	36.67 (36.33, 37.06)	<.001
AKI, n (%)				<.001
No	272 (33.29)	238 (36.96)	34 (19.65)	
Yes	545 (66.71)	406 (63.04)	139 (80.35)	
Hypertension, n (%)				.001
No	426 (52.14)	317 (49.22)	109 (63.01)	
Yes	391 (47.86)	327 (50.78)	64 (36.99)	
Diabetes, n (%)				.483
No	598 (73.19)	475 (73.76)	123 (71.10)	
Yes	219 (26.81)	169 (26.24)	50 (28.90)	
Heart failure, n (%)				.071
No	688 (84.21)	550 (85.40)	138 (79.77)	
Yes	129 (15.79)	94 (14.60)	35 (20.23)	
Myocardial infarction, n (%)				.139
No	793 (97.06)	628 (97.52)	165 (95.38)	
Yes	24 (2.94)	16 (2.48)	8 (4.62)	
Malignant tumors, n (%)				.004
No	744 (91.06)	596 (92.55)	148 (85.55)	
Yes	73 (8.94)	48 (7.45)	25 (14.45)	
Stroke, n (%)				.137
No	766 (93.76)	608 (94.41)	158 (91.33)	
Yes	51 (6.24)	36 (5.59)	15 (8.67)	
Atrial fibrillation, n (%)				<.001
no	653 (79.93)	532 (82.61)	121 (69.94)	
yes	164 (20.07)	112 (17.39)	52 (30.06)	
Obesity er, n (%)				.004
No	717 (87.76)	554 (86.02)	163 (94.22)	
Yes	100 (12.24)	90 (13.98)	10 (5.78)	
Respiratory failure, n (%)				<.001
No	507 (62.06)	425 (65.99)	82 (47.40)	
Yes	310 (37.94)	219 (34.01)	91 (52.60)	
Sepsis, n (%)				<.001
No	300 (36.72)	262 (40.68)	38 (21.97)	
Yes	517 (63.28)	382 (59.32)	135 (78.03)	
CRRT, n (%)				<.001
No	731 (89.47)	605 (93.94)	126 (72.83)	
Yes	86 (10.53)	39 (6.06)	47 (27.17)	
Beta-blocker, n (%)				.520
No	405 (49.57)	323 (50.16)	82 (47.40)	
Yes	412 (50.43)	321 (49.84)	91 (52.60)	
Metformin, n (%)				.220
No	794 (97.18)	623 (96.74)	171 (98.84)	
Yes	23 (2.82)	21 (3.26)	2 (1.16)	
Octreotide, n (%)				.006
No	750 (91.80)	600 (93.17)	150 (86.71)	
Yes	67 (8.20)	44 (6.83)	23 (13.29)	
Statin, n (%)				.382
No	752 (92.04)	590 (91.61)	162 (93.64)	
Yes	65 (7.96)	54 (8.39)	11 (6.36)	
Vasopressin, n (%)				<.001
No	684 (83.72)	585 (90.84)	99 (57.23)	
Yes	133 (16.28)	59 (9.16)	74 (42.77)	
Ventilation, n (%)				.192
No	191 (23.38)	157 (24.38)	34 (19.65)	
Yes	626 (76.62)	487 (75.62)	139 (80.35)	
Gallstones, n (%)				.020
No	663 (81.15)	512 (79.50)	151 (87.28)	
Yes	154 (18.85)	132 (20.50)	22 (12.72)	
Liver disease, n (%)				<.001
No	659 (80.66)	537 (83.39)	122 (70.52)	
Yes	158 (19.34)	107 (16.61)	51 (29.48)	

Continuous variables are expressed as median (IQR), and categorical variables are expressed as number (%).

AKI = acute kidney injury, ALT = alanine aminotransferase, APS III = acute physiology score III, AST = aspartate transaminase, CRRT = continuous renal replacement therapy, DBP = diastolic blood pressure, MBP = mean blood pressure; SpO_2_, percutaneous arterial oxygen saturation, OASIS = oxford acute severity of illness score, PT = prothrombin time, PTT = partial thromboplastin time, RBC = red blood cell, RDW = red cell distribution width, SAPS II = simplified acute physiology score II, SBP = systolic blood pressure, SOFA = sequential organ failure assessment, WBC = white blood cell.

Table 2 presents the baseline characteristics of patients grouped according to quartiles of the AST/ALT ratio. Compared to other groups, *Q*4 was younger. They also showed more derangements in laboratory values (e.g., higher RDW, anion gap, PT, PTT, and creatinine, and lower white blood cell, RBC, platelets, hemoglobin, albumin, sodium, and calcium) and more abnormal vital signs (higher heart and respiratory rates and lower systolic blood pressure). Severity scores (SOFA, APS III, SAPS II, OASIS) were higher in *Q*4, consistent with their greater incidence of acute complications like AKI, respiratory failure, sepsis, and liver disease. *Q*4 participants experienced extended hospital and ICU stays, along with increased in-hospital, ICU, and 365-day mortality.

**Table 2:** Baseline characteristics of the AP population according to AST/ALT ratio quartiles.

	Total (n = 817)	AST/ALT ratio				*P*
		*Q*1 (n = 203)	*Q*2 (n = 204)	*Q*3 (n = 205)	*Q*4 (n = 205)	
Age, yr	59.00 (47.00, 73.00)	64.00 (50.00, 78.00)	61.00 (48.00, 76.00)	60.00 (48.00, 72.00)	52.00 (42.00, 65.00)	<.001
Gender, n (%)						.578
Female	335 (41.00)	78 (38.42)	86 (42.16)	80 (39.02)	91 (44.39)	
Male	482 (59.00)	125 (61.58)	118 (57.84)	125 (60.98)	114 (55.61)	
Weight, kg	83.10 (70.70, 99.00)	83.90 (71.85, 102.25)	84.81 (71.42, 98.62)	82.70 (70.00, 97.30)	80.00 (70.00, 97.50)	.556
WBC, K/µL	12.50 (8.50, 17.90)	13.20 (9.25, 17.25)	13.75 (9.10, 18.80)	12.60 (8.70, 18.10)	10.80 (7.30, 16.00)	.005
RBC, m/µL	3.67 (3.15, 4.20)	3.92 (3.31, 4.41)	3.74 (3.25, 4.19)	3.58 (3.19, 4.16)	3.44 (2.84, 3.97)	<.001
Platelet, K/µL	185.00 (129.00, 258.00)	192.00 (146.50, 249.50)	201.00 (143.75, 275.25)	194.00 (131.00, 293.00)	157.00 (97.00, 215.00)	<.001
Hemoglobin, g/dL	11.20 (9.70, 12.80)	11.90 (10.05, 13.50)	11.20 (10.00, 12.40)	11.20 (9.60, 12.90)	10.80 (9.00, 12.50)	.001
RDW, %	14.50 (13.60, 15.80)	14.30 (13.60, 15.15)	14.40 (13.47, 15.70)	14.50 (13.50, 15.60)	14.80 (13.90, 16.90)	<.001
Albumin, g/dL	3.00 (2.60, 3.30)	3.10 (2.70, 3.40)	3.10 (2.70, 3.32)	2.90 (2.50, 3.30)	2.90 (2.40, 3.20)	<.001
Sodium, mmol/L	138.00 (135.00, 142.00)	139.00 (136.00, 141.50)	139.00 (136.00, 142.00)	138.00 (135.00, 142.00)	137.00 (134.00, 141.00)	.046
Potassium, mmol/L	4.00 (3.60, 4.50)	4.00 (3.70, 4.40)	4.00 (3.60, 4.40)	4.00 (3.60, 4.50)	4.00 (3.60, 4.60)	.747
Calcium, mg/dL	8.00 (7.40, 8.50)	8.10 (7.70, 8.50)	8.10 (7.60, 8.60)	7.90 (7.40, 8.50)	7.50 (6.90, 8.30)	<.001
Chloride, mmol/L	104.00 (100.00, 109.00)	104.00 (101.00, 108.00)	105.00 (101.00, 109.00)	104.00 (100.00, 110.00)	103.00 (99.00, 109.00)	.117
Glucose, mg/dL	125.00 (102.00, 168.00)	127.00 (104.50, 165.50)	130.00 (105.75, 175.50)	123.00 (100.00, 167.00)	121.00 (95.00, 167.00)	.226
Anion gap, mmol/L	15.00 (13.00, 18.00)	14.00 (13.00, 17.00)	15.00 (13.00, 17.00)	15.00 (13.00, 18.00)	17.00 (13.00, 21.00)	<.001
PT, s	14.40 (12.90, 16.70)	14.10 (12.85, 15.70)	14.25 (12.97, 15.72)	14.39 (12.80, 17.30)	14.90 (13.00, 18.90)	.002
PTT, s	30.90 (27.30, 35.90)	30.10 (26.70, 33.95)	30.16 (26.80, 34.23)	30.80 (27.60, 35.50)	32.30 (28.50, 41.00)	<.001
Urea nitrogen, mg/dL	19.00 (12.00, 33.00)	18.00 (12.00, 30.00)	18.00 (11.00, 30.00)	19.00 (11.00, 31.00)	21.00 (12.00, 43.00)	.129
Creatinine, mg/dL	1.00 (0.70, 1.70)	1.00 (0.70, 1.50)	1.00 (0.70, 1.50)	1.00 (0.70, 1.60)	1.30 (0.70, 2.60)	.029
SOFA score	5.00 (2.00, 8.00)	4.00 (2.00, 7.00)	4.00 (2.00, 7.00)	5.00 (2.00, 7.00)	7.00 (4.00, 11.00)	<.001
APS III score	46.00 (33.00, 64.00)	41.00 (31.00, 55.50)	42.00 (31.75, 56.25)	46.00 (33.00, 67.00)	57.00 (41.00, 77.00)	<.001
SAPS II score	34.00 (24.00, 45.00)	31.00 (23.00, 42.00)	32.00 (21.75, 43.00)	33.00 (25.00, 4 6.00)	38.00 (27.00, 53.00)	<.001
OASIS score	32.00 (26.00, 39.00)	29.00 (24.00, 38.00)	31.00 (25.00, 36.00)	32.00 (27.00, 39.00)	35.00 (28.00, 42.00)	<.001
Heart rate, beats/min	97.00 (82.00, 114.00)	93.00 (80.00, 110.50)	95.00 (81.00, 111.25)	100.00 (83.00, 115.00)	101.00 (87.00, 115.00)	.007
SBP, mm Hg	126.00 (109.00, 144.00)	130.00 (113.00, 148.00)	132.00 (111.00, 146.00)	123.00 (108.00, 144.00)	121.00 (104.00, 138.00)	.002
DBP, mm Hg	72.00 (59.00, 85.00)	73.00 (62.00, 88.00)	73.50 (62.75, 87.00)	71.00 (58.00, 84.00)	69.00 (56.00, 84.00)	.176
MBP, mm Hg	85.00 (72.00, 99.00)	85.00 (75.00, 101.00)	86.00 (75.00, 99.25)	85.00 (71.00, 99.00)	82.00 (68.00, 96.00)	.078
Respiratory rate, breath/min	20.00 (16.00, 25.00)	19.00 (16.00, 23.50)	20.00 (16.00, 24.00)	20.00 (17.00, 26.00)	21.00 (17.00, 25.00)	.058
SpO_2_, %	96.00 (94.00, 99.00)	96.00 (94.00, 98.00)	97.00 (94.75, 99.00)	96.00 (94.00, 99.00)	97.00 (95.00, 99.00)	.359
Temperature, °C	36.89 (36.56, 37.28)	36.89 (36.56, 37.22)	36.89 (36.56, 37.33)	36.89 (36.56, 37.39)	36.83 (36.44, 37.22)	.434
AKI, n (%)						.009
No	272 (33.29)	83 (40.89)	65 (31.86)	72 (35.12)	52 (25.37)	
Yes	545 (66.71)	120 (59.11)	139 (68.14)	133 (64.88)	153 (74.63)	
Hypertension, n (%)						.150
No	426 (52.14)	101 (49.75)	100 (49.02)	104 (50.73)	121 (59.02)	
Yes	391 (47.86)	102 (50.25)	104 (50.98)	101 (49.27)	84 (40.98)	
Diabetes, n (%)						.128
No	598 (73.19)	142 (69.95)	141 (69.12)	156 (76.10)	159 (77.56)	
Yes	219 (26.81)	61 (30.05)	63 (30.88)	49 (23.90)	46 (22.44)	
Heart failure, n (%)						.101
No	688 (84.21)	164 (80.79)	172 (84.31)	169 (82.44)	183 (89.27)	
Yes	129 (15.79)	39 (19.21)	32 (15.69)	36 (17.56)	22 (10.73)	
Myocardial infarction, n (%)						.637
No	793 (97.06)	199 (98.03)	196 (96.08)	200 (97.56)	198 (96.59)	
Yes	24 (2.94)	4 (1.97)	8 (3.92)	5 (2.44)	7 (3.41)	
Malignant tumors, n (%)						.145
No	744 (91.06)	178 (87.68)	184 (90.20)	192 (93.66)	190 (92.68)	
Yes	73 (8.94)	25 (12.32)	20 (9.80)	13 (6.34)	15 (7.32)	
Stroke, n (%)						.226
No	766 (93.76)	192 (94.58)	193 (94.61)	186 (90.73)	195 (95.12)	
Yes	51 (6.24)	11 (5.42)	11 (5.39)	19 (9.27)	10 (4.88)	
Atrial fibrillation, n (%)						.158
No	653 (79.93)	164 (80.79)	153 (75.00)	164 (80.00)	172 (83.90)	
Yes	164 (20.07)	39 (19.21)	51 (25.00)	41 (20.00)	33 (16.10)	
Obesity, n (%)						.803
No	717 (87.76)	175 (86.21)	178 (87.25)	181 (88.29)	183 (89.27)	
Yes	100 (12.24)	28 (13.79)	26 (12.75)	24 (11.71)	22 (10.73)	
Respiratory failure, n (%)						<.001
No	507 (62.06)	136 (67.00)	144 (70.59)	115 (56.10)	112 (54.63)	
Yes	310 (37.94)	67 (33.00)	60 (29.41)	90 (43.90)	93 (45.37)	
Sepsis, n (%)						.007
No	300 (36.72)	77 (37.93)	84 (41.18)	84 (40.98)	55 (26.83)	
Yes	517 (63.28)	126 (62.07)	120 (58.82)	121 (59.02)	150 (73.17)	
CRRT, n (%)						<.001
No	731 (89.47)	192 (94.58)	195 (95.59)	186 (90.73)	158 (77.07)	
Yes	86 (10.53)	11 (5.42)	9 (4.41)	19 (9.27)	47 (22.93)	
Beta-blocker, n (%)						.134
No	405 (49.57)	98 (48.28)	94 (46.08)	97 (47.32)	116 (56.59)	
Yes	412 (50.43)	105 (51.72)	110 (53.92)	108 (52.68)	89 (43.41)	
Metformin, n (%)						.840
No	794 (97.18)	197 (97.04)	200 (98.04)	198 (96.59)	199 (97.07)	
Yes	23 (2.82)	6 (2.96)	4 (1.96)	7 (3.41)	6 (2.93)	
Octreotide, n (%)						<.001
No	750 (91.80)	199 (98.03)	192 (94.12)	191 (93.17)	168 (81.95)	
Yes	67 (8.20)	4 (1.97)	12 (5.88)	14 (6.83)	37 (18.05)	
Statin, n (%)						.005
No	752 (92.04)	179 (88.18)	182 (89.22)	194 (94.63)	197 (96.10)	
Yes	65 (7.96)	24 (11.82)	22 (10.78)	11 (5.37)	8 (3.90)	
Vasopressin, n (%)						<.001
No	684 (83.72)	186 (91.63)	182 (89.22)	171 (83.41)	145 (70.73)	
Yes	133 (16.28)	17 (8.37)	22 (10.78)	34 (16.59)	60 (29.27)	
Ventilation, n (%)						.045
No	191 (23.38)	58 (28.57)	54 (26.47)	40 (19.51)	39 (19.02)	
Yes	626 (76.62)	145 (71.43)	150 (73.53)	165 (80.49)	166 (80.98)	
Gallstones, n (%)						<.001
No	663 (81.15)	141 (69.46)	160 (78.43)	180 (87.80)	182 (88.78)	
Yes	154 (18.85)	62 (30.54)	44 (21.57)	25 (12.20)	23 (11.22)	
Liver disease, n (%)						<.001
No	659 (80.66)	187 (92.12)	184 (90.20)	162 (79.02)	126 (61.46)	
Yes	158 (19.34)	16 (7.88)	20 (9.80)	43 (20.98)	79 (38.54)	
Length of stay in hospital, d	16.65 ± 16.96	14.40 ± 15.02	15.13 ± 17.02	17.85 ± 15.57	19.18 ± 19.53	.013
Length of stay in ICU, d	6.31 ± 9.52	5.11 ± 9.59	5.02 ± 8.12	6.52 ± 8.43	8.57 ± 11.25	<.001
Hospital mortality, n (%)						<.001
No	721 (88.25)	190 (93.60)	182 (89.22)	186 (90.73)	163 (79.51)	
Yes	96 (11.75)	13 (6.40)	22 (10.78)	19 (9.27)	42 (20.49)	
ICU mortality, n (%)						<.001
No	755 (92.41)	197 (97.04)	194 (95.10)	190 (92.68)	174 (84.88)	
Yes	62 (7.59)	6 (2.96)	10 (4.90)	15 (7.32)	31 (15.12)	
365-d mortality, n (%)						<.001
No	644 (78.82)	174 (85.71)	162 (79.41)	170 (82.93)	138 (67.32)	
Yes	173 (21.18)	29 (14.29)	42 (20.59)	35 (17.07)	67 (32.68)	

AST/ALT ratio quartiles: *Q*1: 0.22–0.90, *Q*2: 0.90–1.33, *Q*3: 1.33–1.98, and *Q*4: 1.98–9.20.

Continuous variables are expressed as median (IQR), and categorical variables are expressed as number (%).

AKI = acute kidney injury, ALT = alanine aminotransferase, APS III = acute physiology score III, AST = aspartate transaminase, CRRT = continuous renal replacement therapy, DBP = diastolic blood pressure, ICU = intensive care unit, MBP = mean blood pressure, SpO_2_ = percutaneous arterial oxygen saturation, OASIS = oxford acute severity of illness score, PT = prothrombin time, PTT = partial thromboplastin time, RBC = red blood cell, RDW = red cell distribution width, SAPS II = simplified acute physiology score II, SBP = systolic blood pressure, SOFA = sequential organ failure assessment, WBC = white blood cell.

### 3.2. Study outcomes

**Figure 2.**
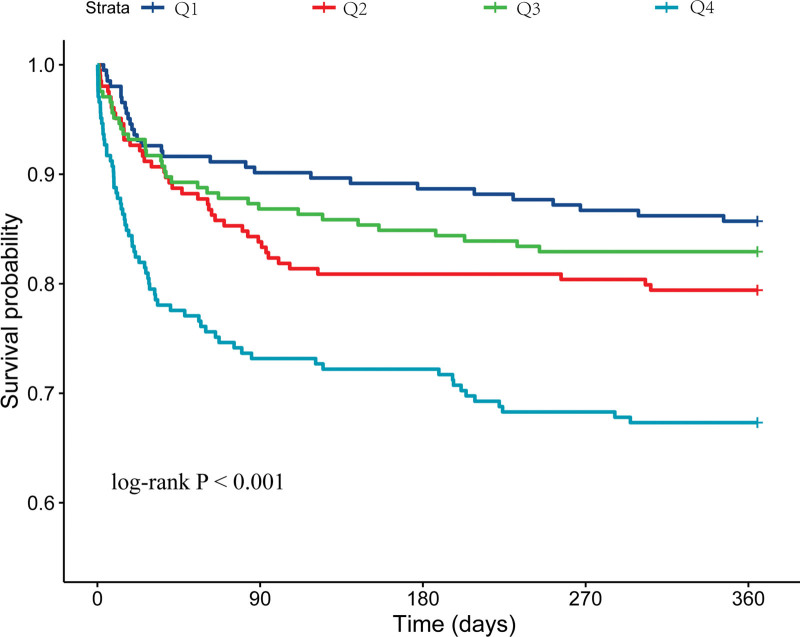
Kaplan–Meier analysis for the association between AST/ALT ratio and 365-day mortality in critically ill patients with AP. AST/ALT ratio, aspartate aminotransferase to alanine aminotransferase ratio. The range for *Q*1 was 0.22–0.90, for *Q*2 was 0.90–1.33, for *Q*3 was 1.33–1.98, and for *Q*4 was 1.98–9.20. ALT = alanine aminotransferase, AP = acute pancreatitis, AST = aspartate aminotransferase.

Kaplan–Meier survival analysis revealed a significant variation in 365-day mortality across AST/ALT ratio quartiles (log-rank *P* < .001) as shown in Figure 2. Patients in *Q*1 had a lower mortality risk compared to those in *Q*4.

Next, multivariate Cox regression analysis found that a higher AST/ALT ratio was an independent factor for 365-day mortality in AP patients (hazard ratio [HR] = 1.33, 95% confidence interval [CI]: 1.16–1.52; Table 3). In addition, after dividing the population into AST/ALT ratio quartiles, patients with *Q*4 had a significantly higher 365-day mortality in fully adjusted model 3, using the *Q*1 population as a reference (HR = 2.11, 95% CI: 1.30–3.40).

**Table 3:** The relationship between AST/ALT ratio and 365-day mortality in critically ill AP patients.

	Model 1		Model 2		Model 3	
	HR (95% CI)	*P*	HR (95% CI)	*P*	HR (95% CI)	*P*
AST/ALT	1.36 (1.23–1.50)	<.001	1.62 (1.45–1.80)	<.001	1.33 (1.16–1.52)	<.001
AST/ALT quartiles						
*Q*1	1.00		1.00		1.00	
*Q*2	1.50 (0.93–2.41)	.094	1.55 (0.97–2.49)	.069	1.58 (0.97–2.56)	.066
*Q*3	1.23 (0.75–2.01)	.416	1.42 (0.86–2.32)	.166	1.09 (0.66–1.81)	.728
*Q*4	2.63 (1.70–4.07)	<.001	3.91 (2.50–6.12)	<.001	2.11 (1.30–3.40)	.002
*P* for trend		<.001		<.001		.011

Model 1: no adjustment.

Model 2: adjusted for age, and weight.

Model 3: adjusted for age, weight, WBC, platelet, RDW, albumin, potassium, calcium, glucose, anion gap, SOFA score, hypertension, diabetes, HLP, gallstones, and liver disease.

AP = acute pancreatitis AST/ALT ratio = aspartate aminotransferase to alanine aminotransferase ratio, CI = confidence interval, HR = hazard ratio, RDW = red cell distribution width, SOFA = sequential organ failure assessment, WBC = white blood cell.

**Figure 3.**
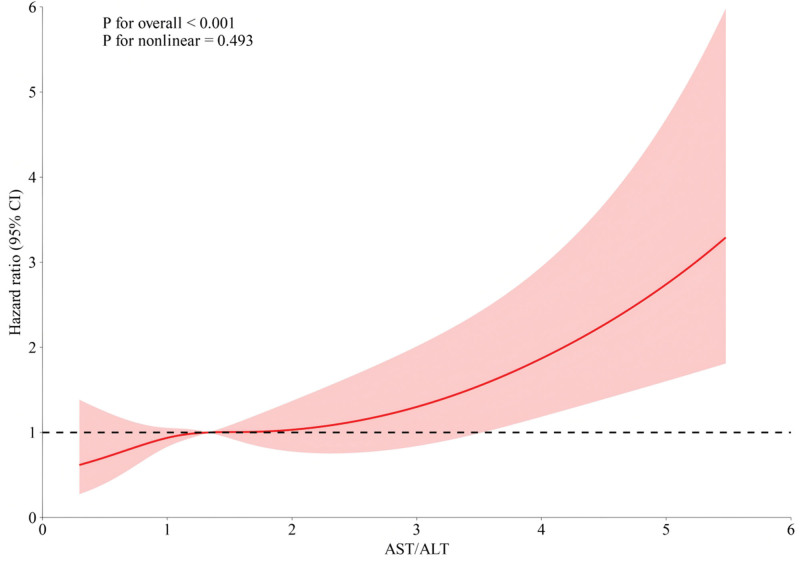
RCS plot of AST/ALT ratio versus 365-day mortality in critically ill patients with AP. AST/ALT ratio, aspartate aminotransferase to alanine aminotransferase ratio. Covariates were adjusted according to model 3. ALT = alanine aminotransferase, AP = acute pancreatitis, AST = aspartate aminotransferase, RCS = restricted cubic splines.

RCS analyses indicated a linear association between the AST/ALT ratio and 365-day mortality, after adjusting for covariates, with a significant overall *P*-value of .001 and a non-significant *P*-value for nonlinear (*P* > .05; Fig. 3).

### 3.3. Subgroup analysis

**Figure 4.**
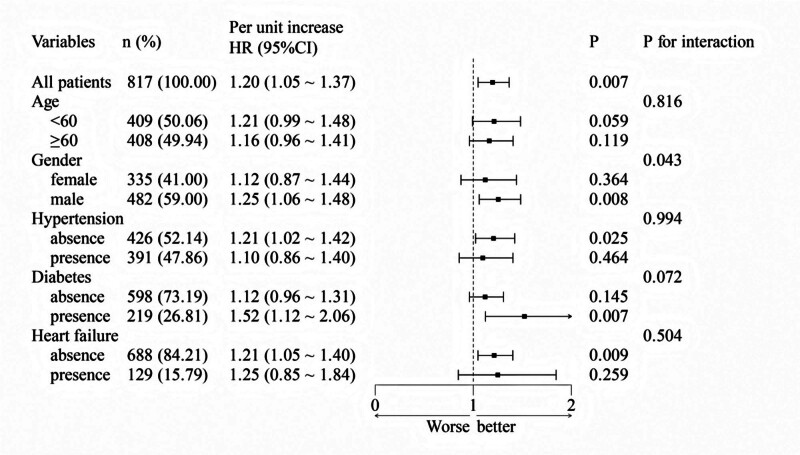
Subgroup analysis between AST/ALT ratio and 365-day mortality in critically ill patients with AP. AST/ALT ratio, aspartate aminotransferase to alanine aminotransferase ratio. Covariates were adjusted according to model 3. ALT = alanine aminotransferase, AP = acute pancreatitis, AST = aspartate aminotransferase.

**Figure 5.**
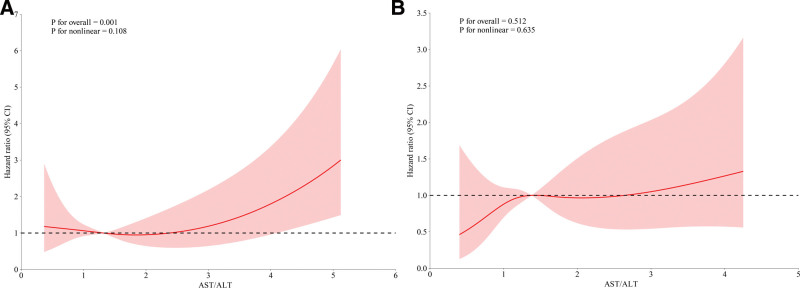
RCS plot of AST/ALT ratio versus 365-day mortality in critically ill patients with AP (A) male, (B) female. AST/ALT ratio, the ratio of aspartate aminotransferase to alanine aminotransferase. Relevant variables were adjusted according to model 3. ALT = alanine aminotransferase, AP = acute pancreatitis, AST = aspartate aminotransferase, RCS = restricted cubic splines.

We conducted a subgroup analysis to examine the protective effect of the AST/ALT ratio in AP patients with varying characteristics (Fig. 4). The positive association between AST/ALT and mortality persisted within all subgroups examined (by age, gender, presence of hypertension, diabetes, and heart failure), supporting the robustness of the finding. In addition, the relationship between the AST/ALT ratio and prognosis in patients with AP varies by gender (*P* for interaction = .043). The association between AST/ALT levels and AP in critically ill patients was more pronounced in males (male: HR = 1.25, 95% CI: 1.06–1.48; female: HR = 1.21, 95% CI: 0.87–1.44). Furthermore, the RCS showed that the association between the AST/ALT ratio and 365-day mortality in patients with AP was more pronounced in males (*P* for overall = .001) and not significant in women (*P* for overall = .512; Fig. 5).

### 3.4. The incremental predictive value of AST/ALT ratio

**Figure 6.**
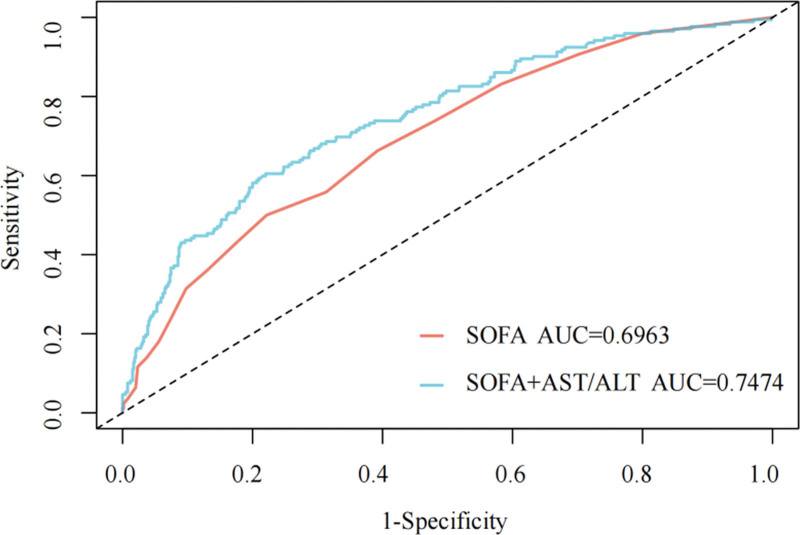
ROC curves for predicting 365-day mortality of critically ill patients with AP. AP = acute pancreatitis, ROC = receiver operating characteristic.

Figure 6 demonstrates the improved predictive performance of adding the AST/ALT ratio to SOFA on the prognosis of patients with AP. Specifically, the inclusion of AST/ALT ratio levels significantly improved the predictive performance of SOFA for 365-day mortality in patients with AP, increasing the area under the curve from 0.6963 to 0.7474.

## 4. Discussion

The current study is the first to explore the prognostic value of the AST/ALT ratio in critically ill patients with AP. After adjusting for potential confounders, a significant positive association was observed between the AST/ALT ratio and 365-day mortality in this patient population. Specifically, each unit increase in the AST/ALT ratio was associated with a 33% higher risk of 365-day mortality in critically ill AP patients. Furthermore, the AST/ALT ratio demonstrated a consistent correlation with mortality across all predefined subgroups of critically ill AP patients. Notably, the integration of the AST/ALT ratio into the SOFA score significantly enhanced its predictive accuracy for mortality in patients with AP, suggesting a potential role as a complementary biomarker in risk assessment.

The AST/ALT ratio serves as a key biomarker for liver health and has garnered attention for its role in reflecting disease risk and prognosis across multiple systems. Recent studies have underscored its significance in digestive, cardiovascular, and endocrine disorders, with particular emphasis on its association with liver pathologies. For instance, a large-scale US study of 4753 participants investigated the inverse ratio (ALT/AST) and reported a positive correlation with nonalcoholic fatty liver disease risk, revealing a striking inverted U-shaped relationship in women.^[34]^ Another US cohort study highlighted that an elevated AST/ALT ratio is predictive of adverse nonalcoholic fatty liver disease prognosis and heightened disease-related mortality.^[35]^ In cardiovascular research, elevated AST/ALT ratios in acute coronary syndrome patients have been linked to increased risks of adverse events and mortality.^[36]^ A study by Matthias et al analyzing 1355 acute myocardial infarction patients demonstrated that the AST/ALT ratio strongly predicted long-term mortality, with each standard deviation increase in the ratio associated with a 23% higher mortality risk.^[37]^ Beyond hepatology and cardiology, the AST/ALT ratio has also been implicated in diabetes onset and related metabolic conditions.^[32,38,39]^ Our study extends these findings by establishing a correlation between the AST/ALT ratio and prognosis in critically ill patients with AP. Given its accessibility in routine clinical practice, the AST/ALT ratio may serve as a valuable tool for prognostic risk stratification in this high-risk patient population.

The study demonstrates a positive correlation between the AST/ALT ratio and 365-day mortality in patients with AP, which may be attributed to the underlying pathophysiology where an elevated AST/ALT ratio reflects severe hepatocellular injury. Hepatocytes contain 2 isoenzymes of AST: cytoplasmic (sAST) and mitochondrial (mAST). In cases of mild hepatocellular damage, only sAST is released into the bloodstream. However, severe hepatocellular injury leads to the release of both sAST and mAST, with serum AST levels rising proportionally to the degree of cellular damage.^[40]^ In patients with severe AP, systemic inflammatory response and oxidative stress are key mechanisms contributing to organ dysfunction and mortality. An influx of inflammatory cytokines into the liver via the portal circulation creates a proinflammatory and immunosuppressive environment, triggering hepatocellular apoptosis and necrosis, which results in hepatic injury and an elevated AST/ALT ratio. Elevated AST levels may be associated with mitochondrial damage and necrotic cell death, whereas ALT release is more indicative of hepatocyte injury. Consequently, an increased AST/ALT ratio may signal more severe liver damage and a more pronounced inflammatory response. Additionally, a high AST/ALT ratio may be linked to the development of MODS. As a central metabolic and detoxification organ, liver dysfunction can disrupt the function of other organs. For instance, hepatic insufficiency may lead to endotoxemia, coagulopathy, and immunosuppression, all of which can worsen the clinical course of AP.^[41,42]^

Subgroup analyses revealed a more pronounced positive correlation between the AST/ALT ratio and 365-day mortality in men. A large-scale study involving over 4,00,000 adults indicated that men were more likely than women to have elevated AST levels (≥40 IU/L).^[43]^ This disparity may be attributed to higher androgen levels in males. Androgens, such as testosterone, promote skeletal muscle protein synthesis and cell proliferation by activating the androgen receptor signaling pathway.^[44,45]^ Since AST is not only present in the liver but also highly expressed in cardiac and skeletal muscle, increased muscle mass may lead to greater AST release. Moreover, men typically consume more alcohol, which can directly cause hepatocellular injury and elevate the AST/ALT ratio.^[46,47]^ Additionally, gender differences in metabolic syndrome may influence liver enzyme profiles. Women are more prone to elevated ALT associated with fatty liver, while men may exhibit a more significant increase in AST due to active muscle metabolism.^[48-50]^ However, the complex interplay between sex and AST/ALT ratios necessitates further investigation through larger and prospective studies to better understand these associations.

In our analysis, when the AST/ALT ratio was treated as a continuous variable, a linear relationship with 365-day mortality was observed. However, when stratified by quartiles, the HR for *Q*2 exceeded that of *Q*3, deviating from monotonicity, and no significant differences were found between *Q*2, *Q*3, and *Q*1. This pattern may stem from the limited sample size. Notably, our study demonstrated that incorporating the AST/ALT ratio significantly improved the SOFA score’s prognostic accuracy for AP patients. As a readily available laboratory marker, an elevated AST/ALT ratio is linked to adverse outcomes in AP and can provide clinicians with immediate prognostic insights upon ICU admission. This enables timely implementation of targeted interventions, including hepatoprotective strategies, metabolic support, and anti-inflammatory measures, particularly for patients presenting with a high AST/ALT ratio.

The current retrospective cohort study, while leveraging a large sample size and adjusting for common confounders, is subject to several limitations. First, the reliance on historical data imposed constraints on variable control during data collection. For instance, critical variables such as imaging findings (e.g., the extent of pancreatic necrosis) and specific treatments (e.g., necrosectomy interventions) were not included, which may limit the ability to draw causal inferences. Second, despite adjusting for known confounders, unmeasured or inadequately controlled variables (e.g., etiology of pancreatitis) could still influence the interpretation of the results. Third, the study could not distinguish whether the prognostic signal of the AST/ALT ratio stemmed directly from pancreatic disease severity or from damage to other organs. Future research should control for baseline liver function data to better isolate the specific impact of AST/ALT on AP progression. Fourth, the AST/ALT ratio was measured only within the first 24 hours of ICU admission. Given the dynamic nature of AP and potential fluctuations in liver enzyme levels throughout the disease course, a single early measurement may fail to capture prognostically relevant trends or peaks. Subsequent studies should incorporate trajectory analysis to explore how dynamic changes in the AST/ALT ratio affect patient outcomes. Fifth, while the MIMIC-IV database offers comprehensive mortality data, it may contain missing data on long-term post-discharge follow-up. This potential misclassification could affect the results, underscoring the need for more robust follow-up data in future research. Sixth, the exclusion of ineligible AP patients may introduce selection bias and limit the generalizability of the findings. Finally, the cohort derived from a single-center database may not represent the full spectrum of AP patients, particularly those not requiring ICU admission. External validation in diverse cohorts is essential to confirm the validity and generalizability of the AST/ALT ratio as a prognostic marker for AP.

## 5. Conclusions

In critically ill AP patients, a higher AST/ALT ratio on admission was associated with greater 365-day mortality, especially in men, suggesting that AST/ALT ratios could be a useful prognostic marker in this particular population.

## Author contributions

**Conceptualization:** Zhimin Dong.

**Data curation:** Zhimin Dong.

**Formal analysis:** Zhimin Dong.

**Investigation:** Zhimin Dong.

**Methodology:** Zhimin Dong.

**Project administration:** Zhimin Dong.

**Resources:** Zhimin Dong, Dasheng Dang.

**Software:** Zhimin Dong.

**Supervision:** Zhimin Dong, Dasheng Dang.

**Validation:** Zhimin Dong, Dasheng Dang.

**Visualization:** Zhimin Dong, Dasheng Dang.

**Writing – original draft:** Zhimin Dong, Dasheng Dang.
